# Development of a novel hydroxylated recombinant human type III collagen TD-1-HrHC in *Komagataella phaffii* with transdermal and skin damage repair activity

**DOI:** 10.1186/s12934-026-03039-1

**Published:** 2026-06-11

**Authors:** Yangyang Li, Yehai Wang, Jingjing Yue, Yan Fan, Shimei Qi, Haixia Yu, Funa Zhang, Jing Wu, Weihua Xiao

**Affiliations:** 1 Institute of Advanced Technology, University of Science and Technology of China, Hefei, China; 2 Anhui Engineering Research Center of Recombinant Protein Pharmaceutical Biotechnology, Hefei, China; 3 State Key Laboratory of Immune Response and Immunotherapy, Hefei, 230027 Anhui China; 4 Hefei Delicate Biotechnology Co., Ltd., Hefei, China; 5 Hefei Cardier Biotechnology Co., Ltd., Hefei, China

**Keywords:** Collagen, Transdermal peptide, *Komagataella phaffii*, Recombinant protein, Protein carbonylation, Skin damage repair

## Abstract

**Background:**

Collagen has been proven to have significant potential applications value in the fields of on medical aesthetics and cosmetics. However, due to its large molecular weight, collagen is difficult to effectively penetrate the skin barrier and reach the designated location to exert the expected activities. Transdermal peptide TD-1 is a short peptide consisting of only 11 amino acids, which can assist proteins such as insulin and cytokines to penetrate the skin barrier and reach the dermis layer. To maximize the transdermal and the efficacy of Collagen III in skincare, the shortest functional fragment that containing a triple helix structure of collagen protein fused with TD-1 were designed and evaluated for its transdermal activity, safety and efficacy.

**Results:**

The recombinant protein containing transdermal TD-1 fragment and a core active fragment of human collagen III (TD-1-HrHC) was co-expressed together with specific hydroxylase using the *K. phaffii* expression system, the products were purified by hydrophobic interaction chromatography (HIC) and ion-exchange chromatography (IEC). The TD-1-HrHC contains about 11.89% hydroxylation modifications that are similar to the natural human collagen III. The transdermal efficiency is 2077% measured by trans-well tests compare to the nature Collagen III isolated from animals. In addition, TD-1-HrHC able to promotes skin damage repairing through regulation series of barrier factors and differentiation factors in the HaCat cell model and promotes the repair of damaged skin and induces the generation of new-born collagen in mouse model. Moreover, it also shown an excellent ability to inhibit protein carbonylation on HaCat cells.

**Conclusion:**

This study demonstrates that TD-1-HrHC can effectively cross the skin barrier and has profound ability to inhibit protein carbonylation of skin and promote skin damage repairing.

**Supplementary Information:**

The online version contains supplementary material available at 10.1186/s12934-026-03039-1.

## Introduction

Collagens are the main structural protein and are widely distributed in the connective tissues of vertebrates and play an indispensable role in various biological functions, such as maintaining normal physiological functions, repairing injuries, and affecting cell attachment and proliferation [1, 2]. Their long half-life, mechanical strength, ability to assemble into fibrils and networks as well as their biocompatibility make collagens be an attractive material in biomedicine and food industries as well as cosmetic products [3–6].

All collagen molecules feature a triple helical domain composed of three α-chains with repeating Gly-X–Y sequences, where X and Y positions commonly contain proline residues [7]. The imino acids proline is often 4-hydroxylated by post-translational modification of the collagen polypeptide chain in order to stabilize the triple-helical structure of collagen [8]. Prolyl 4-hydroxylase (P4H) is a key enzyme in all collagen synthesis. It can catalyze the hydroxylation of proline residues in Gly-X-Pro sequences to form 4-hydroxyproline [9, 10].

In human skin, collagen forms a critical structural component between the epidermis and dermis [3]. Age-related collagen depletion contributes directly to wrinkle formation, loss of elasticity, and other aging phenotypes [11–14]. Beyond its structural role, collagen demonstrates moisturizing properties and facilitates UV damage repair while mitigating oxidative stress, making its supplementation a viable anti-aging strategy [14].

Type III collagen, predominantly synthesized during infancy and termed "baby collagen," undergoes progressive reduction in adulthood [3]. This collagen subtype forms the primary supportive network between skin layers, where its degradation initiates structural collapse [15]. While cosmetic applications increasingly target type III collagen, the skin's barrier function inherently limits macromolecular absorption, necessitating effective delivery systems for bioactive collagen penetration.

Exogenous molecules exceeding 500 Da exhibit markedly reduced stratum corneum permeability [16]. Conventional chemical penetration enhancers (CPEs)—including surfactants, fatty acids, and azone derivatives—show limited efficacy for macromolecules while often causing skin irritation [17]. Without physical augmentation methods like iontophoresis [18], ultrasound [19], or microinjection [20], CPEs cannot deliver compounds above 4.5 kDa through intact skin [16]. These techniques additionally carry operator-dependent risks of tissue damage, highlighting the need for safer collagen delivery solutions. Transdermal peptide (TD-1), is a polypeptide containing 11 amino acids (Ala-Cys-Ser-Ser-Ser-Pro-Ser-Lys-His-Cys -Gly), it can assist macromolecules such as insulin and cell growth factors to cross the skin barrier to reach the dermis by specifically binding to the Na^+^/K^+^-ATPase β subunit (ATP1B1) on the membrane of keratinocytes in the skin, it transiently and reversibly opens the tight junctions between keratinocytes, allowing large-molecule drugs to bypass the skin and rapidly diffuse through the intercellular spaces into the deep layers of the skin [21, 22]. It has excellent activity through the skin epidermis.

In this study, we engineered a hydroxylated recombinant human collagen incorporating both a transdermal TD-1 peptide and a type III collagen bioactive core fragment, expressed in P4H-transformed *K. phaffii* to preserve native hydroxylation patterns. The resulting TD-1-HrHC complex demonstrates human-like hydroxylation modifications, effective transdermal delivery, and significant capacity for inhibit protein carbonylation of skin and promote skin damage repairing, demonstrating substantial translational potential.

## Materials and methods

### Materials

The expression vectors pGAPzα-rDNA and pGAPK were constructed and preserved by our laboratory. Horseradish peroxidase (HRP)-conjugated anti-His tag antibody and anti-P4HB antibody were purchased from Abcam. The *Komagataella phaffii* strain X33 and the *Escherichia coli* strain TOP10 were purchased from Invitrogen (Carlsbad, USA). The human immortalized keratinocyte cell line HaCaT was obtained from DSMZ. All cell lines were cultured in modified RPMI 1640 medium (HyClone, Cat. No. SH30809.01) supplemented with 10% fetal bovine serum (FBS, Biological Industries, Beit HaEmek, Israel), 100 U/mL penicillin, and 100 µg/mL streptomycin (Sangon Biotech (Shanghai) Co., Ltd.), and maintained in a 37 °C incubator with 5% CO₂. All cell lines were authenticated by short tandem repeat (STR) profiling performed by Genewiz (Suzhou, China).

### Molecular design, construction of expression vectors, and screening for expression

The amino acid sequence of the core functional fragment of human type III collagen α1 chain (that is, the amino acid sequence at positions 597–777 in the collagen domain, theoretical molecular weight is 18 kDa, this fragment contains integrin recognition sites, such as “GAOGER” and “GMOGER”, enabling the recombinant collagen to promote cell adhesion and migration, thereby facilitating wound healing. In addition, it enhances the transcriptional activity of collagen-related genes in cells, promoting collagen expression. Furthermore, this fragment retains a substantial number of highly adhesive segments (GER), which effectively promote cell adhesion and activate platelets through the receptors GP Ia/IIa (α2β1), GP VI, and the GP Ib/IX/V complex, thereby accelerating wound healing [23, 24]) was selected, the amino acid residue sequence of transdermal peptide TD-1 (ACSSSPSKHCG) was connected at the N-terminal, and the coding gene sequence of KEX2 protease cleavage site was introduced at the front end of the TD-1 sequence. An amino acid sequence (6 × His) connected at the C end; Following codon optimization, Xhol and NotⅠ restriction enzyme sites were added to the 5ʹ and 3ʹ ends of the fusion gene, respectively. The full-length sequence was synthesized by a company (Genewiz) and inserted into the Zeocin-resistant vector pGAPzα-rDNA, resulting in the construction of the protein expression vector pGAPzα-rDNA-TD-1-HrHC. The construction and screening of the expression strain were performed with reference to previous studies [25].

### Fermentation production and downstream processing

Based on our previous study [25], the fermentation of recombinant strains was conducted according to the fermentation guidelines provided by Invitrogen. The selected high-expression strain was inoculated into a shaking tube containing 4 mL of YPD medium and cultured in a shaking incubator at 30 °C for 24–48 h until the OD600 reached 2–6, yielding the primary seed culture for fermentation. One milliliter of this seed culture was then transferred into a conical flask containing 200 mL of BMGY medium and cultured in a shaking incubator at 30 °C for 12–24 h to obtain the secondary seed culture. The entire 400 mL of secondary seed culture was inoculated into a fermenter containing 6 L of BMGY medium supplemented with an additional 2 g/L trisodium citrate. The fermentation parameters were set as follows: cultivation temperature 28 °C, pH 6.0, with continuous monitoring of dissolved oxygen and agitation speed. During the cell growth phase, BMGY medium was used. When a rapid increase in dissolved oxygen was observed, indicating depletion of the basal glycerol in the medium, glycerol feeding was initiated at a rate of 70 mL/h. The fermentation temperature was then adjusted to 25 °C and maintained until the end of fermentation. After fermentation, the culture broth was centrifuged at 10,000×*g* for 30 min to collect the supernatant, which was subsequently clarified by filtration through 0.45 μm and 500 kDa microfiltration columns using a FlexStand system (GE Healthcare Life Sciences, Chicago, USA).

Subsequently, hydrophobic interaction chromatography and ion-exchange chromatography were performed sequentially to further purify TD-1-HrHC. Hydrophobic Interaction Chromatography (Phenyl Bestarose (HS), Catalog No. AH003, Bestchrom):1. Sample Preparation: The fermentation supernatant following solid–liquid separation was prepared. Ammonium sulfate was added to a final concentration of 30% (176 g per liter of supernatant), stirred until dissolved, and filtered through a 0.22 μm in-process fiber filter. 2. Equilibration: The column was washed with equilibration buffer (20 mM PB + 243 g/L ammonium sulfate) for at least 5 column volumes. 3. Loading: The prepared sample was loaded under specified conditions at a linear flow rate of 250–400 cm/h. 4. Wash: The column was washed with equilibration buffer (20 mM PB + 243 g/L ammonium sulfate) until the UV absorbance returned to near baseline. 5. Elution: Elution was performed using 20 mM PB. The eluate was collected upon a sharp increase in UV absorbance, and collection was terminated when the absorbance began to decline gradually. Ion Exchange Chromatography (CM Bestarose FF, Catalog No. AI0044, Bestchrom): (1) Sample Preparation: The eluate from hydrophobic interaction chromatography was desalted/diluted to a conductivity of no more than 5 mS/cm, and the pH was adjusted to 5.0. (2) Equilibration: The column was washed with equilibration buffer (50 mM acetate/acetic acid buffer, pH 5.0) until the pH and conductivity of the outlet buffer were consistent with the equilibration buffer, typically requiring 3–5 bed volumes. (3) Loading: The prepared sample was loaded under specified conditions at a linear flow rate of 60–300 cm/h. (4) Equilibration: The column was washed with equilibration buffer (50 mM acetate/acetic acid buffer, pH 5.0) until the pH and conductivity of the outlet buffer were consistent with the equilibration buffer, typically requiring 3–5 bed volumes. 5. Elution: Elution was performed using 20 mM PB + 0.5 M NaCl. The eluate was collected upon a sharp increase in UV absorbance, and collection was terminated when the absorbance began to decline gradually. The eluate was desalted, and the buffer was exchanged to 20 mM PB.

The purified protein was analyzed by SDS-PAGE and HPLC (TSKgel G2000SWXL) to assess the purity of the recombinant protein. The mobile phase used was 1 × PBS buffer (pH 6.0). The purified protein complex was then filtered through a 0.22 μm membrane, aliquoted, and stored at − 80 °C.

### Hydroxyproline content determination

Take 1 mL of the sample to be tested, add equal volume of concentrated hydrochloric acid, and hydrolyze at 110 ℃ for 6 h. After the reaction was completed, the supernatant was centrifuged at 12,000×*g* for 20 min (room temperature), the supernatant was completely collected, the pH value of the supernatant was adjusted to 6.0–8.0, and then the sample solution to be measured was obtained with ultra-pure water. According to the instructions of the kit (Hydroxyproline Detection Kit, mlbio, ml092985), the color reaction was configured. After 20 min of color development at 60 ℃ and 15 min of room temperature equilibrium, the data were collected and analyzed with the help of an enzyme-labeled instrument.

### Transdermal performance test of TD-1-HrHC in mice and suckling pig

Skin was freshly excised from healthy mice and Suckling pigs immediately after sacrifice. Subcutaneous fat was removed by blunt dissection with extreme care to avoid any mechanical damage to the epidermal surface. Each skin sample was carefully inspected under a bright light by at least two trained personnel. Any sample exhibiting even minor scratches, holes, or uneven thickness was discarded. Only skins with a visually flawless, uniform surface were selected. Then, the skin from healthy mice and Suckling pigs was fixed between the supply chamber and the receiving chamber of the diffusion tank, with the cuticle facing the supply chamber and the dermis facing the receiving chamber. Add 6.5 ml 1 × PBS to the receiving chamber as the receiving solution, add the sample (2000 μg/ml, 1 ml) to be measured to the supply chamber, and then start the diffuser. The temperature was maintained at 37 ℃ throughout the process. Collect the receiving chamber solution at the appropriate time (24 h and 48 h or 0 h、 4 h and 24 h) or take skin samples for protein concentration or fluorescence intensity testing.

### Transdermal performance test of TD-1-HrHC in human

The anterior region of the human forearm was selected as the test site for Raman spectroscopic measurements. The skin test area measured 1 × 1 cm2, and measurements were conducted at four time points: 0 h, 0.5 h, 4, and 12 h. A single subject was enrolled, and three test areas, each measuring 1 × 1 cm2, were delineated. Within each test area, five points were selected for parallel measurements, with three repeated measurements performed at each point. The test compound was applied via topical administration, with the dosage determined as 2 mg/cm^2^, in accordance with the standard protocol for in vivo human Raman spectroscopy. Data were acquired using a LabRAM Soleil ultra-high-resolution confocal Raman microscope, and the relative permeation rate of TD-1-HrHC was calculated.

Statistical Methods for Results: Processing of Raman spectroscopic imaging data comprised two main components: spectral preprocessing and data analysis. Spectral preprocessing included removal of cosmic rays, spectral smoothing, background noise removal, baseline correction, and spectral normalization. Univariate data analysis focused on the Raman spectroscopic analysis of biochemical substances corresponding to specific peak positions, thereby untangling their distribution within human skin. Data analysis was performed using Labspec software for baseline correction and identification of characteristic peak positions. The obtained Raman spectra were then calculated for parameters including peak intensity, peak shift, peak area, and full width at half maximum (FWHM). Concurrently, Labspec software was employed to perform numerical analysis of peak intensities at varying skin depths and to generate spatial distribution maps.

The permeation behavior of the sample was determined based on its characteristic Raman signals, which are distinct from the intrinsic skin signals, thereby identifying its spatial distribution at different depths within the skin.

### Detection of anti-carbonylation ability of TD-1-HrHC

#### Model construction

Biochemical level:

Configure the reaction system: (The reaction system was completed to 1 mL with PBS).

ROS pathway:

**Table:** 

Reagent	Testing group	Negative control group	Positive control group
BSA	15 mg/mL	15 mg/mL	15 mg/mL
FeSO_4_	200 μM	200 μM	200 μM
EDTA	200 μM	200 μM	200 μM
H_2_O_2_	10 mM	10 mM	10 mM
TD-1-	1/5/10 μM	–	–
HrHC	–	–	10 μM
GSH			

Incubation at 37 ℃ for 24 h in the dark;

RCS pathway:

**Table:** 

Reagent	Testing group	Negative control group	Positive control group
BSA	15 mg/mL	15 mg/mL	15 mg/mL
MDA	3 mM	3 mM	3 mM
TD-1-	1/5/10 μM	–	–
HrHC	–	–	10 μM
GSH			

Incubation at 37℃ for 24 h in the dark;

Then, the degree of protein carbonylation was detected by DNPH colorimetry.

At the cellular level:

Cell model construction 100 mm petri dish was used, 15 mL medium was added, and the initial inoculation amount was 1 × 10^5^ cells /mL for 72 h. Experimental group: Blank control group: HaCaT cells cultured with DMEM medium; Negative control group: HaCaT cells were cultured in DMEM medium containing 80 mM glucose. Positive control group: HaCaT cells were cultured in DMEM medium containing 80 mM glucose, and GSH was added to the medium to make the final concentration is 10 μM. TD-1-HrHC-low testing group: HaCaT cells were cultured in DMEM medium containing 80 mM glucose, and TD-1-HrHC was added to the medium to make the final concentration is 1 μM. TD-1-HrHC-mid testing group: HaCaT cells were cultured in DMEM medium containing 80 mM glucose, and TD-1-HrHC were added to the medium, so that the final concentration was 5 μM. TD-1-HrHC-hig testing group: HaCaT cells were cultured in DMEM medium containing 80 mM glucose, and TD-1-HrHC were added to the medium, the final concentration of which was 10 μM. When the cells grew to about 90% confluent, the cells were collected. 600 μL RIPA cracking solution was added and the supernatant was collected after cracking at room temperature for 15 min. Then 67 μL 0.1 g/mL streptomycin sulfate was added and allowed to stand for 10 min, 12,000*g* centrifuge for 5 min, and supernatant was collected. BCA protein quantitative kit was used to detect the protein concentration of each group, and PBS was used to regulate the protein concentration of each group to make it equal. The degree of protein carbonylation was detected by DNPH colorimetry.

The degree of protein carbonylation was determined by DNPH colorimetry.

Remove 100 μL of the sample to be tested from the reaction system, add 400 μL of 10 mM DNPH solution (configured with 2 M HCl), and put the system in a place away from light for 1 h, and swirl and mix it every 10 min. Add 500 μL of 200 g/L trichloroacetic acid, gently mix, stand at 4 ℃ for 5 min to make the reaction complete, centrifuge at 12,000*g* at 4 ℃ for 15 min, discard the supernatant; Precipitation was washed with 1 mL ethanol: ethyl acetate (volume ratio 1:1) and washed three times (washing method: add washing liquid, shake well, centrifuge at 4 ℃, 12,000*g* for 15 min); The precipitation was dissolved in 1.25 mL 6 M guanidine hydrochloride in 37 ℃ water bath for 15–30 min. The mixture of 12,000*g* was centrifuged for 15 min and the supernatant was taken. The absorption value of supernatant was measured at 370 nm wavelength.

Carbonyl content calculation formula:

$$ \begin{aligned} Carbonyl{\text{ }}content\left( {nmol/mgprot} \right) & = \left( {Test{\text{ }}group{\text{ }}OD370{\text{ }}{-}{\text{ }}Blank{\text{ }}control{\text{ }}OD370} \right) \\ & \times \varepsilon \div \left[ {22 \times Path{\text{ }}length\left( {cm} \right) \times Protein{\text{ }}concentration\left( {\mu g/ml} \right)} \right] \\ \end{aligned} $$

Ε: Extinction coefficient of carbonyl group, the value is 1.25 × 10^5^

Path length: 1 cm.

### Skin damage repair experiment in HaCat cell model

6 × 10^4^ HaCat cells were inoculated in a 6-well culture dish. The cells were cultured until a dense monolayer was formed. A 200 μL pipette tip was used to gently mark a straight line from the middle to form damage. Meanwhile, the medium was changed to serum-free DMEM medium containing the sample to be tested. The cells were incubated in a 5% CO_2_ incubator at 37 ℃, and the damage repair conditions were observed at 6 h, 12 h and 24 h, respectively, and were observed by optical microscope and photographed. According to the images recorded at different time points, the repair speed was measured, and the experimental results were statistically analyzed.

Calculation formula of repair rate:

$$ \begin{aligned} Repair{\text{ }}rate{\text{ }}\left( {damage{\text{ }}healing{\text{ }}rate} \right){\text{ }} & = {\text{ }}\left( {initial{\text{ }}damage{\text{ }}area{\text{ }} - {\text{ }}damage{\text{ }}area{\text{ }}at{\text{ }}t{\text{ }}time} \right) \\ {\text{ }} & \div {\text{ }}initial{\text{ }}damage{\text{ }}area \\ \end{aligned} $$

After that, total RNA was extracted from the cells, and the expression of differentiation factors and barrier factors related to skin injury repair was detected by real-time fluorescent quantitative PCR. The corresponding factors and primers sequences were shown in Supplementary Table 1.

### Skin damage repair experiment in mouse model

Male BALB/c mice aged 8 weeks and weighing 20.0 ± 5.0 g were selected to establish a full-thickness skin wound model for evaluating the effect of collagen on wound healing. All mice were housed in a standard laboratory environment with a 12 h/12 h (light/dark) cycle and acclimatized for one week prior to the experiment. One day before the experiment, the dorsal hair of the mice was shaved, and the animals were randomly divided into 4 groups, with 10 mice per group. Anesthesia was induced by intraperitoneal injection of 1.25% tribromoethanol (25 µL/g). Subsequently, a circular full-thickness skin defect wound with a diameter of 4 mm was created on the dorsal skin of each mouse to simulate a wound surface. Wounds in each group were treated with different concentrations of collagen solution, which was applied dropwise and evenly spread using a cotton swab; treatments were administered once every 4 days. The blank control group was treated with an equal volume of physiological saline. On days 0, 4, 8, and 13, 5 mice from each group were selected. The wound area was photographed using a digital camera to document the healing process, and wound area was quantitatively analyzed using Image J software.

### Histological analysis and masson staining

On postoperative days 4, 8, and 13, skin tissue samples were collected from 3 mice per time point and fixed in 10% formalin for over 24 h. The samples were then embedded in paraffin and sectioned into 4 μm thick slices. Sections were stained with Hematoxylin and Eosin (H&E) and Masson's trichrome to observe tissue morphology and collagen distribution, respectively. Collagen density in the stained sections was quantitatively analyzed using Image J software.

### Statistical analysis

Prior to data analysis, the normality of the data distribution was assessed using the D’Agostino-Pearson test. For data that followed a normal distribution, statistical analyses were performed using GraphPad Prism 7.0. The primary methods employed included paired and unpaired two-tailed t-tests, multiple t-tests, and one-way ANOVA. For data that did not conform to a normal distribution, analyses were conducted using IBM SPSS Statistics 22 software, with the primary method being the Wilcoxon signed-rank test. For significance testing, p values > 0.05 were considered not significant (ns); p values < 0.05 were considered significant and marked with *; p values < 0.01 were marked with **; p values < 0.001 were marked with ***; and p values < 0.0001 were marked with ****.

## Results

### Molecular design and expression of hydroxylated TD-1-HrHC

**Fig. 1:**
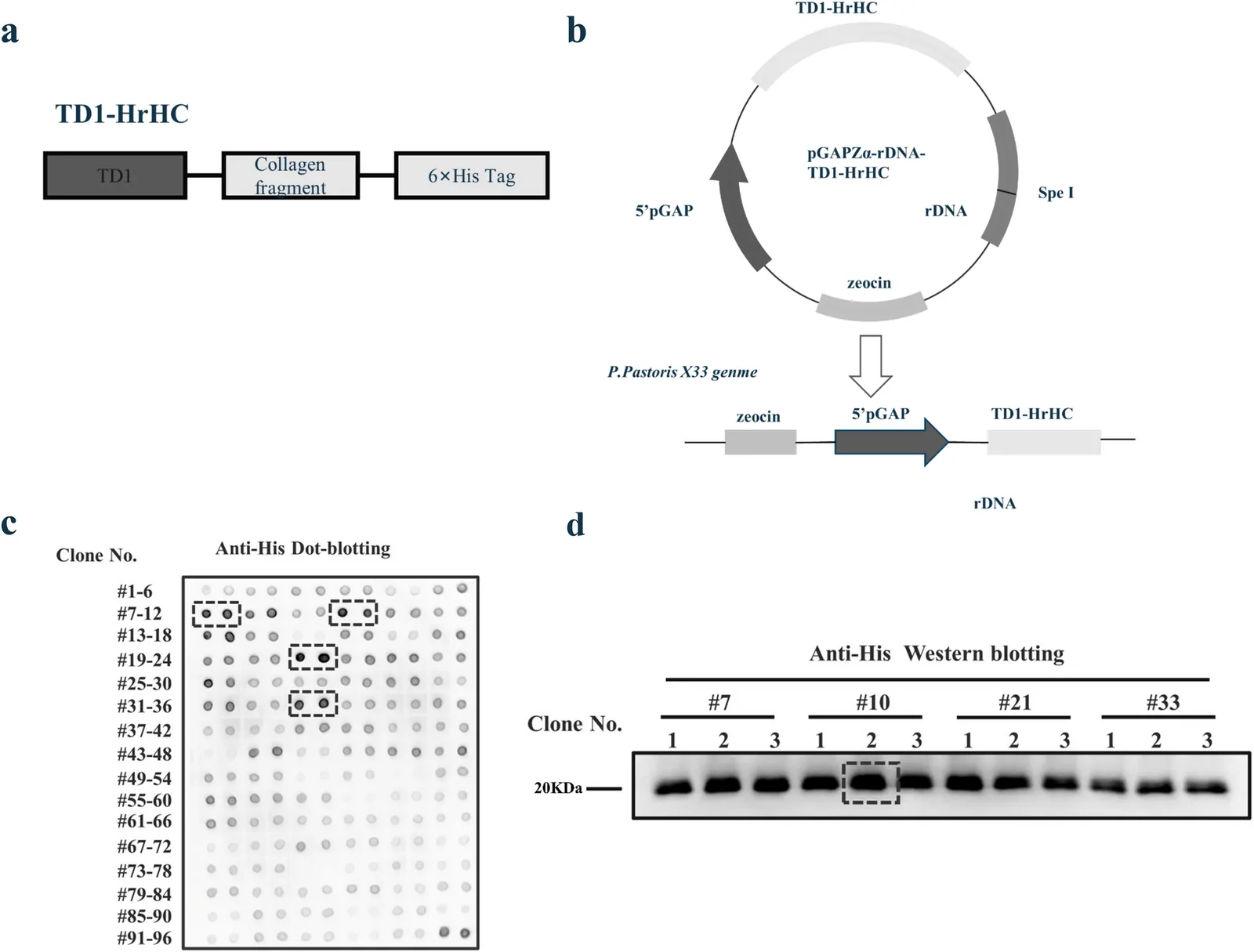
Construction and expression of TD1-HrHC in *K. phaffii*. **a** Schematic representation of the TD1-HrHC molecular structure. The recombinant protein consists of the transdermal peptide TD-1, the core bioactive domain derived from the α1 chain of human type III collagen, and a C-terminal 6 × His tag. **b** The TD1-HrHC coding sequence was cloned into the pGAPzα vector downstream of the constitutive GAP promoter and integrated into the genome of *P. pastoris* X33 strain via homologous recombination. **c** Positive transformants secreting TD1-HrHC were initially screened by dot-blot analysis using an HRP-conjugated anti-His antibody. **d** Candidate clones with elevated expression were further confirmed by Western blotting under non-reducing conditions (without DTT). Clone #10–2 exhibited the strongest signal and was selected for subsequent experiments

Numerous investigations demonstrate that recombinant human collagen can be produced via genetic engineering methods, with rhCOL3A1 displaying identical characteristics to native human COL3A1 [2, 26, 27]. In this study, we cloned and co-expressed the P4H gene, TD-1 gene and the human COL3A1 gene in *K. phaffii* X33 to obtain hydroxylated TD-1-HrHC complex. The P4H was composed of P4Hα and P4Hβ. In order to ensure the normal function of P4Hα and P4Hβ, a KEX2 sequence was added between the expression sequences of P4Hα and P4Hβ (SFig. 1A). The TD-1-HrHC complex was composed of TD-1, the core active fragment of human collagen III and a 6 × His tag at the C-terminal (Fig. 1a). The TD-1 fragment of TD-1-HrHC has good transdermal activity, which can assist the active fragment of collagen through the epidermal layer, effectively improve the efficiency of collagen entering the skin, exert its activity, and help improve the skin condition.

The coding sequences for TD-1-HrHC and P4H were adjusted to match the codon preferences of *K. phaffii*, subsequently synthesized, and ligated into the pGAPzα and pGAPK vectors, respectively (Fig. 1b and SFig. 1B). These vectors integrate into the X33 strain's genome via homologous recombination. The TD-1-HrHC gene was linked to the alpha-factor signal peptide and regulated by the GAP promoter (Fig. 1b).

For developing a high-yield strain of the hydroxylated TD-1-HrHC complex, the plasmids pGAPK-P4H and pGAPzα-TD-1-HrHC were introduced into *K. phaffii* X33 through electroporation, followed by plating on YPD agar supplemented with 1000 µg/ml G418 or 300 µg/ml zeocin. P4H complex high-producers were initially identified via dot blot assays, with promising candidates verified by Western blot. A top performer (#17-2) was chosen for subsequent transformations (SFig. 1C and D). Similarly, hydroxylated TD-1-HrHC high-producers were preliminarily selected by dot blotting and confirmed via Western blotting, with one standout clone (#10–2) advanced to pilot-scale fermentation (Fig. 1c and d).

### Fermentation production, downstream processing and identification of hydroxylated TD-1-HrHC

**Fig. 2:**
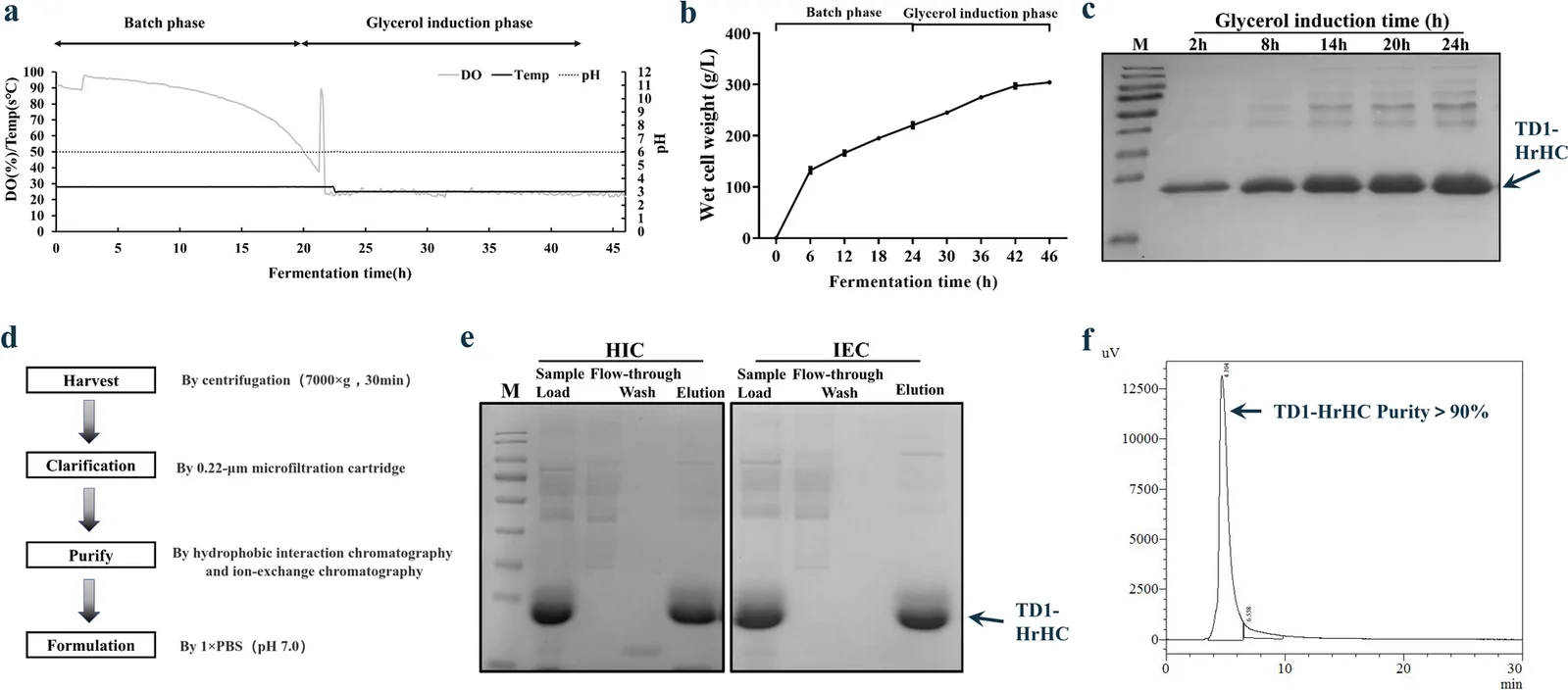
Pilot-scale (10 L) fermentation, purification, and quality assessment of TD1-HrHC. **a** Overview of the optimized two-phase fermentation strategy, comprising an initial batch phase followed by a glycerol-fed induction phase. Key parameters including dissolved oxygen (DO), temperature, and pH were continuously monitored. **b** Changes in wet cell weight (WCW) of the TD1-HrHC-expressing strain at different time points throughout fermentation. **c** Accumulation of secreted TD1-HrHC in the culture supernatant during the glycerol induction phase, analyzed by SDS-PAGE under reducing conditions and visualized by Coomassie Brilliant Blue staining. **d** Flowchart illustrating the downstream purification process of the fermentation supernatant. **e** Assessment of TD1-HrHC purity at each purification step using SDS-PAGE under reducing conditions followed by Coomassie Brilliant Blue staining. **f** Final purity of the target protein determined by high-performance liquid chromatography (HPLC). *M* Pre-stained protein molecular weight marker

We subsequently refined the production conditions for 10 L-scale fermentation to yield substantial quantities of recombinant protein exhibiting the desired biological function. A biphasic fermentation strategy (Fig. 2a) was employed, consisting of an initial batch stage (0–22 h) followed by a glycerol-fed induction stage (22–46 h), with fine-tuned settings for temperature, glycerol concentration, and pH (SFig. 2). Following the batch stage, wet cell weight (WCW) attained 210 g/L, progressively rising to 305 g/L by fermentation's conclusion (n = 5 runs, Fig. 2b). In the induction period with glycerol feeding, TD-1-HrHC levels in the supernatant built up consistently, stabilizing after 20 h (Fig. 2c). By the process's end, densitometry analysis revealed that TD-1-HrHC constituted roughly 80% of the total recombinant proteins in the supernatant (Fig. 2c). The supernatant was harvested via centrifugation and filtration, after which the complexes underwent hydrophobic interaction chromatography (HIC) for enrichment and ion-exchange chromatography (IEC) for additional refinement. Post-HIC and IEC recoveries for TD-1-HrHC stood at 87.30% and 74.70%, respectively, culminating in approximately 34.7 mg/L of purified target protein. The end product was stored in 1 × PBS (pH 7.4) buffer (Fig. 2d, e). Purity of the isolated protein complex was evaluated using SDS-PAGE and HPLC (Fig. 2e, f), with SDS-PAGE indicating exceptional cleanliness for the target, corroborated by HPLC results showing over 90% purity. Subsequently, hydroxylation extent in TD-1-HrHC from three batches was assessed using a specialized protein hydroxylation assay kit, yielding hydroxyproline contents of 11.55%, 12.20%, and 11.92%, respectively—values approximating the 13% hydroxyproline in native type III collagen (Table 1). Collectively, these findings confirm successful production of the anticipated TD-1-HrHC featuring hydroxylation akin to its human counterpart, alongside a reliable manufacturing protocol.

**Table 1:** Results of TD-1-HrHC hydroxylation levels in three different production batches

Production batch	Hydroxyproline content (%)
1	11.55
2	12.20
3	11.92

### TD-1-HrHC has excellent transdermal activity and can effectively penetrate the epidermis of mice and Suckling pigs

**Fig. 3:**
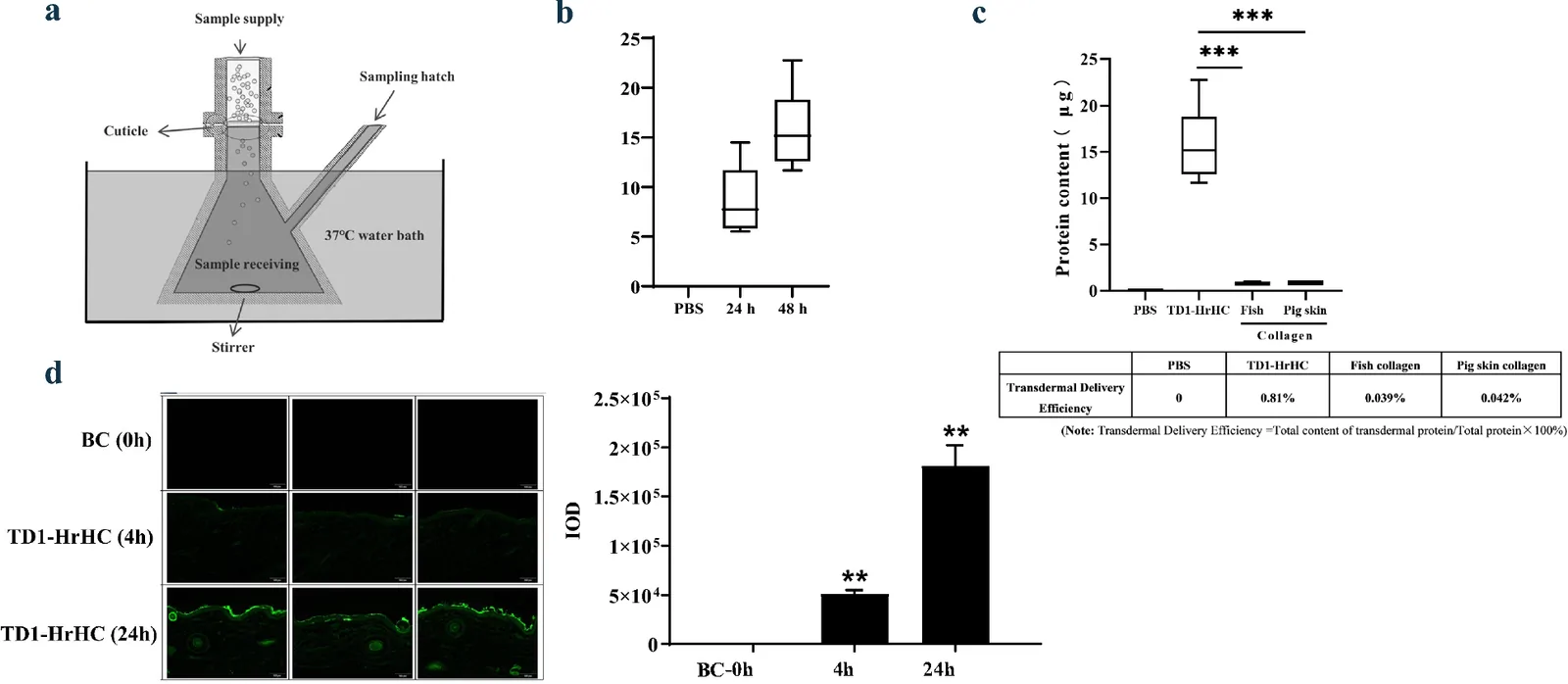
TD1-HrHC has excellent transdermal activity and can effectively penetrate the epidermis of mice and Suckling pigs. **a** Model of transdermal experiment. Skin from healthy mice was placed between the receiving chamber and the diffusion chamber of the transdermal performance test instrument, TD1-HrHC or other collagen were added to the diffusion chamber, and then samples were taken at different time points to detect the concentration changes of the target samples in the receiving chamber. **b** Changes in the concentration of TD1-HrHC protein detected at different time points in the receiving chamber. **c** The results of different protein reaction after 48 h. **d** The Suckling pig skin was placed between the receiving chamber and the diffusion chamber of the transdermal testing instrument, and TD1-HrHC was added to the diffusion chamber, and then the concentration of the TD1-HrHC entering the Suckling pig skin through the fluorescence microscope was observed at different time points. Datas are represented as the mean ± SD from 5 independent experiments. *ns* not significant; *p < 0.05; **p < 0.01; ***p < 0.001

In order to help collagen better cross the skin barrier to achieve its excellent activity, transdermal peptide TD-1 was connected to its N terminal. To verify whether TD-1-HrHC has the expected efficient transdermal activity, mouse skin of appropriate size was placed between the supply chamber and the receiving chamber of the static diffusion cell. During this period, the receiving cell was kept at a constant temperature of 37 ℃ and the liquid level was sufficient. Ensure that the skin is moist, thus simulating human skin for testing the transdermal ability of TD-1-HrHC (Fig. 3a). The results showed that when only PBS was added in the feeding chamber, no protein signal was detected in the receiving chamber, indicating that there were no interfering factors affecting the specificity of the experiment in the system (Fig. 3b and c). When 2000 μg TD-1-HrHC was added into the feeding chamber, the incubation time was 24 h, and the content of TD-1-HrHC in the receiving tank was 8.83 ± 3.39 μg. When the incubation time was increased to 48 h, the content of TD-1-HrHC in the receiving pool increased to 16.08 ± 3.74 μg/mL (Fig. 3b). In addition, we compared TD-1-HrHC with commercially available collagen peptides from fisheries and animal husbandry (fish skin collagen peptide ≤ 3000 Da, pig skin collagen peptide ≤ 750 Da). Only 0.77 ± 0.18 μg of collagen peptide from fish skin and 0.83 ± 0.19 μg/mL from pig skin were penetrated into the receiving pool within 48 h, while the ability of TD-1-HrHC to cross the skin barrier was significantly higher than that of commercially available collagen peptides, about 2077% (Fig. 3c).

In order to further confirm the excellent transdermal activity of TD-1-HrHC, we used Suckling pig skin instead of mouse skin for transdermal experiments, and observed the performance of TD-1-HrHC through the skin with fluorescence microscopy. We found that at the beginning, the fluorescence signal of TD-1-HrHC was not observed in Suckling pig skin, and as time went on, higher and higher fluorescence signal of TD-1-HrHC was detected in Suckling pig skin (Fig. 3d). These results indicate that TD-1-HrHC can effectively penetrate and enter mouse Suckling pig skin.

Furthermore, the percutaneous permeation behavior of TD1-HrHC in human skin was evaluated using Raman spectroscopy. The Raman spectrum of TD1-HrHC is presented in Supplementary Fig. 3A. The characteristic Raman peaks were observed at 348 cm^⁻1^, 487 cm^⁻1^, 541 cm⁻1, 777 cm^⁻1^, 849 cm^⁻1^, 902 cm^⁻1^, 964 cm^⁻1^, 1094 cm^⁻1^, 1306 cm^⁻1^, 1454 cm^⁻1^, 1659 cm^⁻1^, 2735 cm^⁻1^, 2935 cm^⁻1^, 2977 cm^⁻1^, 3225 cm^⁻1^, and 3428 cm^⁻1^. These peaks were subsequently utilized as spectral fingerprint information for tracking the permeation of TD1-HrHC. The full-thickness Raman spectra of human skin obtained 12 h after TD1-HrHC application are shown in Supplementary Fig. 3B. Depth-dependent variations in the peak intensities of keratin and lipids were observed. Through in-depth analysis of the Raman mapping images (Supplementary Fig. 3C), the distribution of TD1-HrHC at various depths within human skin in vivo was obtained. As illustrated in Supplementary Fig. 3C, TD1-HrHC penetrated the stratum corneum within 0.5 h, reached the viable epidermis within 4 h, and approached the dermis by 12 h, indicating its excellent transdermal activity.

Taken together, these results indicate that the TD-1-HrHC has excellent transdermal activity.

### TD-1-HrHC has excellent resistance of protein carbonylation of skin

**Fig. 4:**
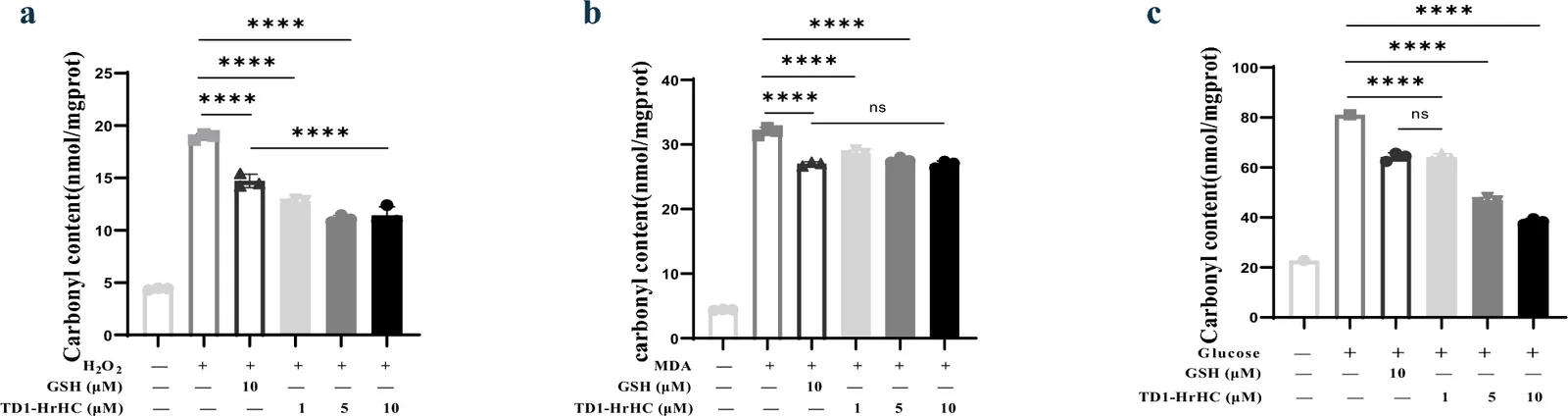
TD1-HrHC has excellent resistance of protein carbonylation of skin. **a** Detection of the carbonylation capacity of TD1-HrHC anti-protein Reactive oxygen species (ROS) pathway (H_2_O_2_-induced construction). **b** Detection of anti-protein lipid peroxidation (RSC) pathway (MDA induced construction) carbonylation ability of TD1-HrHC. **c** Detection of the anti-protein carbonylation ability of TD1-HrHC in HaCaT cells. Datas are represented as the mean ± SD from 5 independent experiments. *ns* not significant; *p < 0.05; **p < 0.01; ***p < 0.001; ****p < 0.0001

Protein carbonylation in the skin occurs primarily through two pathways: reactive oxygen species (ROS)-mediated oxidation and reactive carbonyl species (RCS)-induced lipid peroxidation [28–30]. To model these processes, we treated bovine serum albumin (BSA) with H₂O₂ (simulating ROS oxidation) and malondialdehyde (MDA, simulating lipid peroxidation), using glutathione (GSH) as a positive control to quantify protein carbonylation [31–33]. The model involves oxidative modification of BSA amino acid residues by H₂O₂ or MDA, with carbonyl content serving as an inverse measure of a compound's anti-carbonylation capacity. H₂O₂ treatment elevated BSA carbonyl content from 4.39 ± 0.08 nmol/mg protein (negative control) to 18.94 ± 0.29 nmol/mg protein, while MDA produced more extensive modification (32 nmol/mg protein), confirming successful model establishment. At 10 μM, TD-1-HrHC showed superior anti-carbonylation activity (11.40 ± 0.87 nmol/mg protein) compared to GSH (14.72 ± 0.63 nmol/mg protein) in the ROS pathway model, while exhibiting comparable efficacy to GSH (26.94 ± 0.52 vs. 27.02 ± 0.29 nmol/mg protein) in the MDA-induced model. These results demonstrate that TD-1-HrHC's anti-carbonylation capacity matches or exceeds GSH in both pathways, with dose-dependent effects evident across tested concentrations (Fig. 4a and b).

The high concentration of glucose in the culture environment can also cause the protein in the cell to be carbonylated, resulting in cell senescence [34–36]. Therefore, we added additional glucose (80 mM) to HACAT cell culture medium and cultured it with different concentrations of TD-1-HrHC for 72 h to evaluate the anti-protein carbonylation activity of TD-1-HrHC at the cellular level by detecting the degree of protein carbonylation in cells. The results showed that the carbonyl content of protein increased from 22.8 to 81.1 nmol/mgprot in negative control group. The results of the experiment group showed that TD-1-HrHC had significant intracellular anti-protein carbonylation ability, and the intracellular protein carbonylation level was 64.1 ± 1.2 nmol/mgprot when the concentration was 1 μM. The resistance to intracellular protein carbonylation modification was not less than 10 μM GSH (64.2 ± 1.4 nmol/mgprot) (Fig. 4c). In addition, TD-1-HrHC also showed a dose effect, the higher the concentration, the lower the level of intracellular protein carbonylation.

### TD-1-HrHC has excellent potential to promote skin damage repair and induces the generation of new-born collagen

**Fig. 5:**
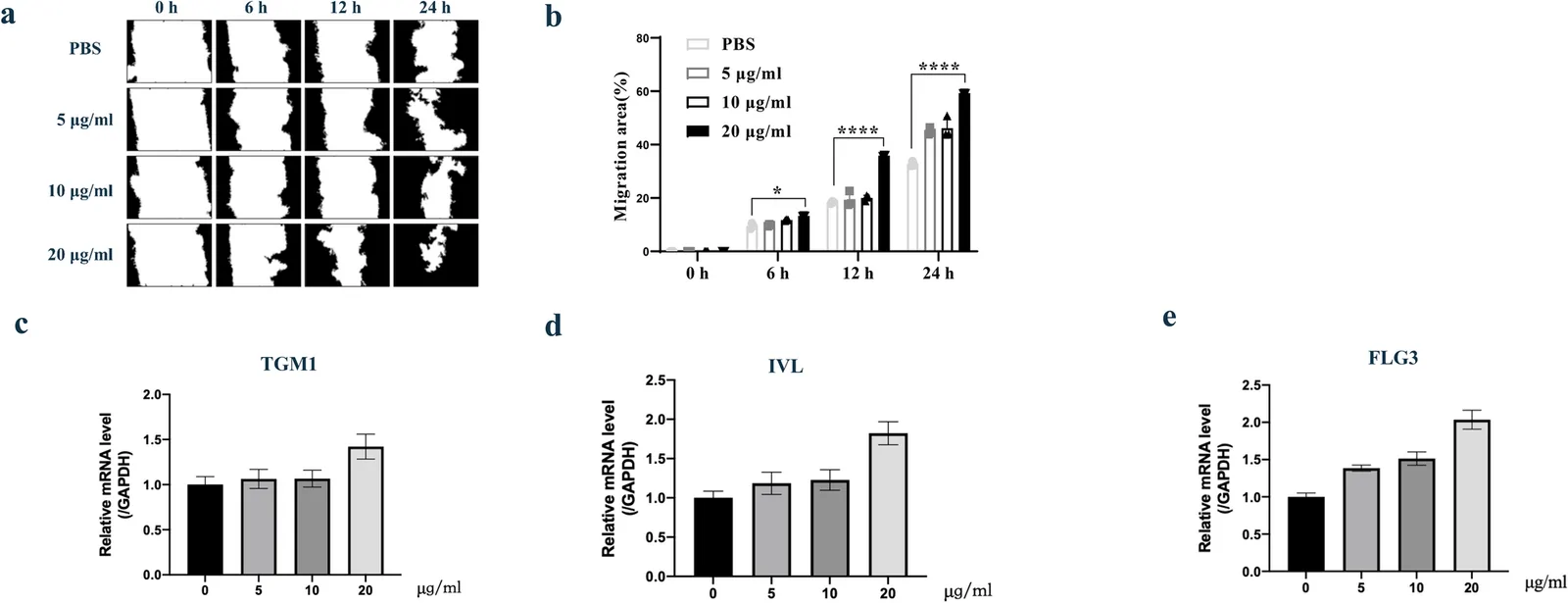
TD1-HrHC has excellent potential to promote skin damage repair. **a** The repair of HaCaT cells at the injury site in serum-free medium with different concentrations of TD1-HrHC at different time points, where black is the area of cell repair and growth, and white is the area of injury and scratch. **b** Change of repair rate over time. c-f: At the end of the damage repair experiment (24 h), the expression of genes related to skin damage repair was detected. **c** Results of FLG3 gene expression after treatment with different concentrations of TD1-HrHC. **d** Results of IVL gene expression after treatment with different concentrations of TD1-HrHC. **e** Detection of TGM1 gene expression after treatment with different concentrations of TD1-HrHC. Datas are represented as the mean ± SD from 5 independent experiments. *ns* not significant. *p < 0.05; **p < 0.01; ***p < 0.001; ****p < 0.0001

Natural collagen has been proved to have a strong ability to promote damage repair, and is widely used in damage protection and skin damage repair [37–40]. In order to verify the ability of TD-1-HrHC could promote damage repair, we examined the scratch repair ability of TD-1-HrHC on human skin keratinocytes. We first marked the central area of the cells that had been attached to the wall and use a micro gun head to make a scratch in the central area, cleaned the cells in the central scratched part with PBS, and continued to culture HaCaT cells with serum-free medium containing the samples to be tested until the time set by the experiment. After the same field of view was taken by the microscope, in order to highlight the cell migration area, the ImageJ software was used to process the results and strengthen the contrast between the cell growth area and the blank area, where the white part was the scratch blank area, and the black part was the cell migration and growth area. The results of data analysis showed that when the cells were cultured in serum-free DMEM medium containing 20 μg/mL TD-1-HrHC for 24 h, the repair rate of the cells reached 59.2 ± 0.9%, which was much higher than that of the negative control group (32.3 ± 0.8%). Moreover, the repair rate of TD-1-HrHC increased with the increase of protein concentration (Fig. 5a and b).

In order to further analyze the underlying mechanism of TD-1-HrHC promoting skin injury repair, we detected the expression levels of barrier factors and differentiation factors closely related to skin injury repair at the RNA level. We found that the expression of differentiation-related factors FLG3, as well as barrier factors IVL and TGM1 were significantly upregulated in keratinocytes treated with TD-1-HrHC, which play a crucial role in skin barrier damage repair. It is suggested that the damage repair ability of the simple skin model treated by TD-1-HrHC has been greatly improved.

**Fig. 6:**
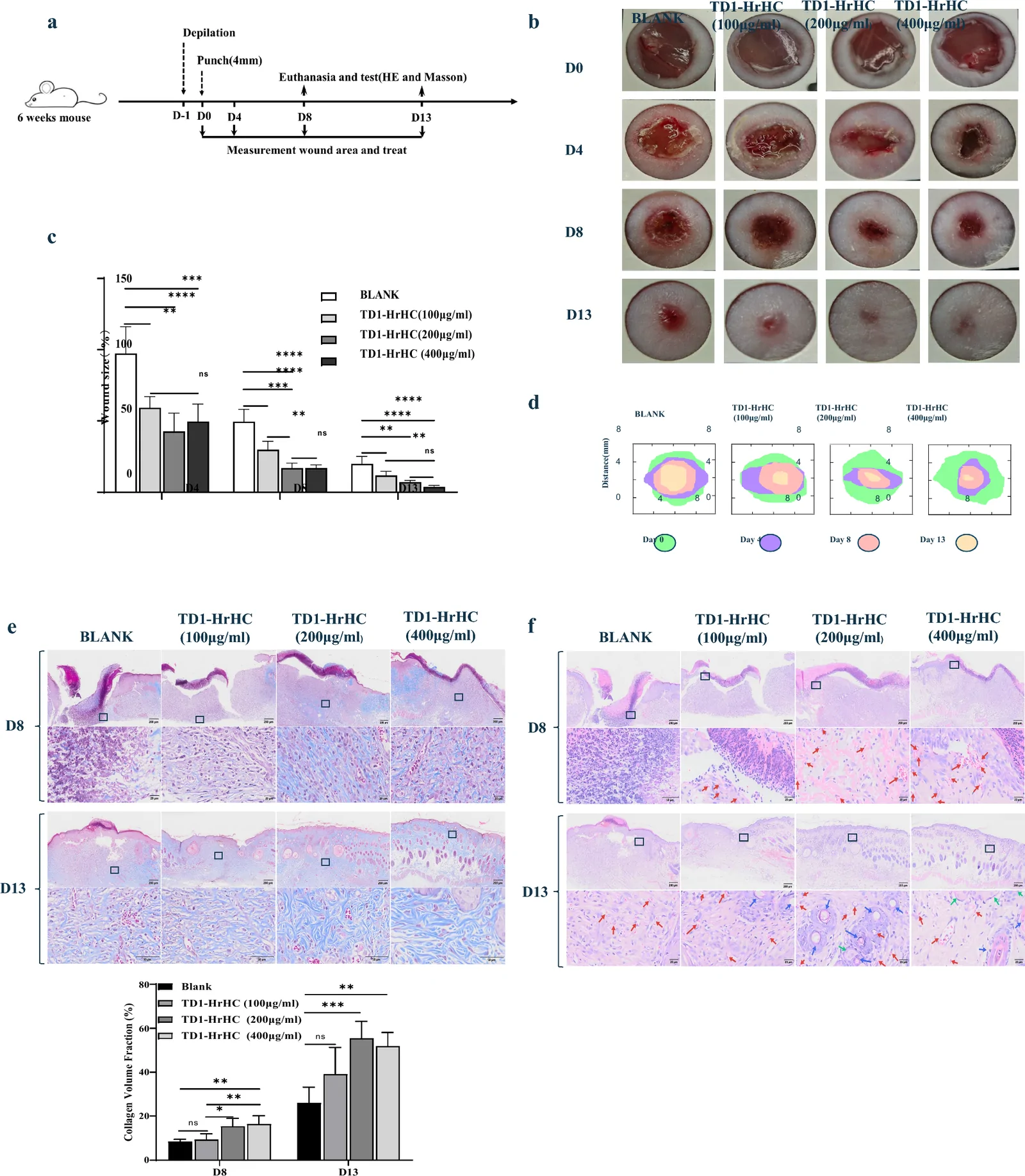
TD1-HrHC effectively promotes the repair of injured skin and induces the generation of new-born collagen. **a** Schematic diagram of the experimental protocol. Ten healthy 6-week-old Balb/c mice were used. One day before the experiment, the dorsal hair of the mice was removed. On the start day of the experiment, uniform wounds were created in the experimental area on the mice using a punch biopsy tool. The wound healing status was assessed at predetermined time points, and different drug treatments were administered. On Day 8 and Day 13, five mice were sacrificed respectively for Hematoxylin and Eosin (HE) staining and Masson's trichrome staining to evaluate skin repair. **b** Wound healing status in mice at different time points. **c** The relative wound size at different time points compared to that on Day 0. **d** Overlay comparison of wound sizes at different time points. **e** Masson’s trichrome staining results of the wound repair area(Top panel) and Collagen content in the wound repair area(Below panel). **f** HE staining results of the wound repair area (Red arrow: blood vessel; Blue arrow: hair follicle; Green arrow: sebaceous gland). *p < 0.05; **p < 0.01; ***p < 0.001; ****p < 0.0001

To further and more intuitively and scientifically evaluate the capacity of TD-1-HrHC in promoting skin damage repair, a mouse model of skin injury was established (Fig. 6a). It was observed that TD-1-HrHC effectively accelerated the repair process of damaged skin in mice, exhibiting a concentration-dependent damage repair effect, which is consistent with the aforementioned findings (Fig. 6b–d). Moreover, TD-1-HrHC not only significantly enhanced the rate of damage repair but also improved the quality of tissue regeneration. Damages treated with TD-1-HrHC did not develop prominent scars, and the disrupted dermal structures associated with skin integrity were restored more rapidly and completely, displaying clearer and more organized morphology (Fig. 6e and f). Additionally, it is noteworthy that TD-1-HrHC-treated damages exhibited increased deposition of newly synthesized collagen, indicating that TD-1-HrHC effectively promotes the synthesis of nascent collagen (Fig. 6e).

Taken together, these results suggest that TD-1-HrHC has excellent activity in promoting skin damage repair.

## Discussion

In this study, we designed a new recombinant human collagen containing a transdermal TD-1 fragment and a core active fragment of human collagen, and selected *K. phaffii* as an expression vector for the production of the protein. In addition, we also transferred P4H gene into yeast to obtain recombinant human collagen with close to human hydroxylation modification, so as to retain the activity of human collagen to the greatest extent. Our study showed that the recombinant human collagen TD-1-HrHC has similar hydroxylation modification to natural human collagen and excellent transdermal activity. In addition, TD-1-HrHC also has excellent ability to inhibit protein carbonylation of skin and promote skin damage repair, which has great practical application value.

Safe and effective source of collagen raw materials is the key to the industrialization of collagen cosmetics [41]. Industrial collagen currently originates primarily from livestock (bovine and porcine byproducts) and fisheries (marine byproduct processing). Bovine and porcine collagen carries zoonotic disease risks, including TSE, BSE, and FMD [41], while marine-derived collagen exhibits inferior stability, reduced hydroxyproline content, and lower denaturation temperatures [42, 43]. Although genetic engineering has enabled recombinant collagen production in Escherichia coli or yeast, challenges persist, including bacterial contamination risks, incomplete proline hydroxylation, and potential methanol residues in yeast-based systems [44–46]. Here, we employed *K. phaffii* as an expression vector, introducing an additional P4H gene and controlling TD-1-HrHC expression via glycerol induction, yielding recombinant human collagen with near-native hydroxylation levels and enhanced safety. This approach offers a viable alternative for cosmetic-grade collagen production.

The ability of skin care products to cross the epidermal barrier and reach the dermis to exert their biological effects serves as a key functional indicator. However, exogenous molecules exceeding 500 Da exhibit markedly reduced penetration through the stratum corneum [16]. Collagen demonstrates substantial potential for medical aesthetic and cosmetic applications [3, 5, 6, 15], yet its high molecular weight impedes effective skin barrier penetration, limiting its broader application. While chemical enhancers and physical delivery methods moderately improve collagen transport efficiency, they remain constrained by skin irritation, suboptimal delivery rates, and technical complexity [17, 18, 20]. The 11-amino acid transdermal peptide TD-1 facilitates dermal penetration of large molecules including insulin and cytokines, exhibiting superior transdermal properties [21]. This peptide may address current limitations in collagen delivery technology. By conjugating the core active fragment of human collagen III with this transdermal peptide, we developed a recombinant human collagen with enhanced penetration capability. In vitro studies demonstrate that our TD-1-HrHC construct effectively traverses animal skin (murine and porcine models), showing significantly greater transdermal efficiency than commercial fishery- and livestock-derived collagen peptides, thereby ensuring optimal skin barrier penetration. Although the absence of instrumental skin integrity assessment (e.g., TEWL or electrical resistance) is a limitation of the present study, all the test samples were tested under the same conditions, so this result still indicates that TD-1-HrHC has an excellent ability to penetrate the skin.

Protein carbonylation involves the introduction of carbonyl groups into amino acid side chains of proteins, serving as a key marker of oxidative damage [30]. This modification occurs when carbonyl groups such as aldehydes, ketones, or lactams incorporate into protein side chains through distinct pathways [28]. As an irreversible post-translational modification, protein carbonylation induces conformational changes and backbone cleavage, ultimately impairing protein function. Cellular protein carbonylation proceeds via two primary mechanisms: direct oxidation by reactive oxygen species (peroxide, superoxide, and hydroxyl radicals) through the ROS pathway [47, 48], or modification by unsaturated aldehydes (MDA and HNE) via the RCS pathway [49, 50]. Keratin in keratinocytes of skin is the main target of protein carbonylation. Human skin, especially facial skin, is often exposed to ultraviolet radiation, which causes reactive oxygen chain reaction to produce ROS, lipid peroxidation to produce a large amount of MDA and HNE, and then protein in keratinocytes of skin is carbonylated [51]. Keratin carbonylation exerts three principal effects on skin physiology. First, it compromises moisture retention, as carbonylated proteins reduce bound water content in the stratum corneum [52]. Second, it diminishes skin elasticity by impairing barrier function, with more pronounced effects observed during autumn and winter [53]. Third, it alters light refraction through keratin fiber restructuring, decreasing skin transparency and producing a dull appearance [54]. Using H_2_O_2_ and MDA to simulate oxidative and peroxidative processes in BSA, we established biochemical and cellular models of protein carbonylation in high glucose-cultured HaCaT cells. These models demonstrated the potent anti-carbonylation activity of TD-1-HrHC, suggesting its promising cosmetic applications.

In contemporary society, human skin encounters numerous stressors, including ultraviolet radiation, environmental pollutants, and excessive cosmetic treatments, all of which contribute to cutaneous damage. Medical procedures such as laser therapy and microneedle may also induce skin trauma [55]. Consequently, there is growing consumer demand for products capable of facilitating effective skin damage repair. Novel restorative products could address this market need by offering enhanced dermatological and cosmetic solutions, potentially gaining competitive commercial advantages. Our findings demonstrate that TD-1-HrHC significantly accelerates damage repair in scratch-induced skin model while upregulating key barrier proteins including TGM1, IVL, FLG3 and in skin damage repair mouse model while promoting the repair of subcutaneous tissue and the synthesis of new-born collagen. These results demonstrated its substantial potential for developing therapeutic agents targeting skin damage repair. However, it must be acknowledged that the mouse skin injury repair model established and utilized in this work has certain limitations, unlike human wound healing, which relies primarily on re-epithelialization and granulation tissue formation, rodent wound closure occurs mainly through contraction of the panniculus carnosus [56]. We did not use a splinting method in this study. And in the future studies should employ splinted models to further validate our findings.

## In summary

In this study, we successfully developed a novel recombinant human collagen product TD-1-HrHC based on the *K. phaffii* expression platform, which can effectively cross the skin barrier, play its excellent anti protein carbonylation and promote skin damage repair activities. These results indicate that TD-1-HrHC has excellent value and potential in the development of new biological cosmetics and medical beauty products.

## Supplementary Information

Additional file 1.

## Data Availability

The datasets used and/or analysed during the current study are included in this article and its supplementary information files, and are available from the corresponding author upon reasonable request. No datasets were generated or analysed during the current study.

## Abbreviations

TD-1: Transdermal peptide 1
*K. phaffii*: *Komagataella phaffii*
HrHC: Hydroxylated recombinant human type III collagen
CPE(s): Chemical penetration enhancer (s)
P4H: Prolyl 4-hydroxylase
WCW: Wet cell weight
PBS: Phosphate buffered saline
TGM1: Transglutaminase 1
IVL: Involucrin
FLG3: Filaggrin 3
