# Supplementary material for: Development of a novel hydroxylated recombinant human type III collagen TD-1-HrHC in Komagataella phaffii with transdermal and skin damage repair activity
Source: Microb Cell Fact. 2026 Jun 11;25:173. doi: 10.1186/s12934-026-03039-1 (PMC13491638; doi:10.1186/s12934-026-03039-1)
Supplement: Supplementary file 1 [file 12934_2026_3039_MOESM1_ESM.docx]

**Supplementary Fig. 1 Construction and expression of recombinant prolyl 4-hydroxylase (P4H) in *K. phaffii*. a.** Schematic illustration of the P4H heterodimer structure. The functional enzyme is formed by co-expression of the P4Hα catalytic subunit and the P4Hβ regulatory subunit. **b.** The bicistronic P4H expression cassette, driven by the constitutive GAP promoter, was cloned into the pGAPK vector and subsequently integrated into the genome of P. pastoris X33 strain via homologous recombination. **c.** Positive transformants secreting the P4H complex were initially identified by dot-blot screening using an HRP-conjugated anti-P4H antibody. **d.** High-producing clones with the strongest signals were further validated by Western blotting under non-reducing conditions (no DTT added) with the same anti-P4H antibody. Among them, clone #17-2 displayed the highest expression level and was therefore chosen for subsequent co-transformation and fermentation studies.

**Supplementary Fig. 2 Optimization of culture conditions of TD1-HrHC engineered strains. a.** Detection of protein expression at different culture temperatures. **b.** Detection of protein expression at different glycerol concentrations. **c.** Detection of protein expression at different culture PH.

**Supplementary Fig. 3 TD1-HrHC can effectively penetrate human skin. a.** Raman spectrum of TD1-HrHC. **b.** Full-thickness Raman spectra of human skin at 12 h post-application of TD1-HrHC. **c.** Raman permeation mapping image of TD1-HrHC in human skin.

**Supplementary Fig.4 Original bright-field microscopy images of the repair of HaCaT cells at the injury site in serum-free medium with different concentrations of TD1-HrHC at different time points**

**Supplementary Fig.5 TD1-HrHC can be preserved stably for extended periods. To evaluate its stability, lyophilized TD1-HrHC was stored under various temperature conditions, and purity changes were monitored at different time points using HPLC. The results of the long-term stability evaluation for lyophilized TD1-HrHC are presented as follows: a.** under room-temperature storage; **b.** under storage at 4 ℃; **c.** under storage at 45 ℃.

**Supplementary Table 1** **The corresponding factors and their primers sequences.**

| **Gene** | **Primer Sequence** | |
| --- | --- | --- |
| **FLG** | Forward Prmer | GACAGTGAGGCATACCCAGAG |
|  | Reverse Primer | AAGTGACCATGTTCCTTAGCG |
| **IVL** | Forward Prmer | TCCTCCAGTCAATACCCATCA |
|  | Reverse Primer | CATTCTTGCTCAGGCAGTCC |
| **TGM1** | Forward Prmer | GGCAAGTTTCAGTTCACAGTCC |
|  | Reverse Primer | GGTCCCGTAGTAAATTCTCCC |
